# Association between hearing loss and frailty: a systematic review and meta-analysis

**DOI:** 10.1186/s12877-021-02274-y

**Published:** 2021-05-25

**Authors:** Rong Tian, Osvaldo P. Almeida, Dona M. P. Jayakody, Andrew H. Ford

**Affiliations:** 1 Medical School, University of Western Australia, 35 Stirling Highway, Western Australia 6009 Perth, Australia; 2 WA Centre for Health & Ageing, University of Western Australia, Perth, Western Australia Australia; 3 Ear Science Institute Australia, Subiaco, Western Australia Australia; 4 Ear Sciences Centre, Faculty of Health and Medical Sciences, University of Western Australia, Perth, Australia

**Keywords:** Age related hearing loss, hearing impairment, physical function, frail elderly

## Abstract

**Background:**

Frailty is associated with poor health outcomes in later life. Recent studies suggested that hearing loss may be a potentially modifiable risk factor associated with frailty.

**Methods:**

This systematic review and meta-analysis aimed to investigate the association between hearing loss and frailty in observational studies of adults aged 50 years or over. We included observational studies with participants ≥ 50 years old that have clear descriptions of hearing and frailty measurement methods. Meta-analyses were conducted using measurement of risk and 95 % confidence interval of each individual study. Quality assessment, risk of bias, heterogeneity and sensitivity analyses were also conducted. Our study followed PRISMA guidelines.

**Results:**

Our search identified 4508 manuscripts published in English between 1 and 2000 and 9 February 2021. Sixteen articles reported acceptable measurements of both hearing loss and frailty. Two papers were not suitable for meta-analysis. Twelve sets of cross-sectional data involving 12,313 participants, and three sets of longitudinal data involving 3042 participants were used in the meta-analysis. Hearing loss was associated with an 87 % increase in the risk of frailty among cross-sectional studies (risk ratio [RR] 1.87; 95 %CI 1.63–2.13) and 56 % among longitudinal studies (RR 1.56; 95 %CI 1.29–1.88). There was considerable heterogeneity among studies, but their quality rating, sample size or approach used to assess hearing loss did not change the results substantially.

**Conclusions:**

The findings of this systematic review and meta-analysis of observational studies suggest that hearing loss increases the risk of frailty in later life. Whether this relationship is causal remains to be determined.

**Supplementary Information:**

The online version contains supplementary material available at 10.1186/s12877-021-02274-y.

## Background

Frailty is a common clinical syndrome in older adults that is associated with adverse health outcomes and increased mortality over time [1]. There is ongoing debate as to how best to define frailty, but most approaches are based on declining body mass, strength, endurance, balance, gait speed, and daily activity [2, 3]. Several well-validated frailty models have been established and many screening tools are available for use in clinical practice and research [1, 4]. One popular approach is based on the model that arose from the Cardiovascular Health Study (Fried criteria). It identifies an individual as frail when three or more of the following criteria are present: unintentional weight loss, self-reported exhaustion, muscle weakness, slow walking speed, and low physical activity [2]. Nonetheless, the lack of consensus on how best to define frailty may have contributed to the rather discrepant prevalence estimates of frailty in later life, ranging from 4 to 59 % [5, 6] in the community and to nearly 70 % among institutionalized older people [7].

Recent research findings have shown that frailty is not a static syndrome, and many of its signs and symptoms can be reversed, at least partly, with appropriate interventions, such as physical activity, diet and review of medications [8–10]. These promising results have led to renewed efforts to uncover other potentially modifiable risk factors associated with frailty, and hearing loss is one of them. Hearing loss has a high prevalence among aged people. Age related hearing loss (ARHL) is the most common cause of hearing loss, affecting 40 % of the population aged 65 years and over [11]. Data from the 2005–2006 National Health and Nutritional Examination Survey in the USA suggested a 63 % prevalence of hearing loss in adults aged 70 years and above [12]. Notwithstanding its high prevalence, the proportion of people with ARHL who receive appropriate treatment remains low [13]. A UK study reported that only one-third of hearing impaired adults older than 70 years who could have benefit from hearing aids actually owned them, and among those with hearing aids, 10 % never used them [14].

There are a number of potential pathways linking hearing loss to frailty [15]. Hearing loss is reported to increase the risk of incident falls in older adults [13, 15, 16], and a significantly great odds of depression has also been observed [17]. Hearing loss has also been associated with poor physical function [18, 19] and reduced activities of daily living [19, 20] among older adults. Panza et al. [21] suggested that age-related hearing loss and frailty, as well as cognition, share pathophysiological pathways, and that the use of hearing devices could potentially alleviate frailty. However, opposing evidence suggesting there is no association between these two also exist [22, 23] and the available evidence is far from conclusive.

Given the negative health outcomes associated with frailty and the high prevalence of hearing loss in this age-group, it seems important to determine if this association is likely to be causal. An initial step in this process would be to determine if there is an association between hearing loss and frailty by reviewing currently available studies investigating this. Thus, this systematic review and meta-analysis aimed to examine the association between hearing loss and frailty in later life. Specifically, we wanted to determine if in older adults aged 50 years or older, does hearing loss increase the risk of frailty compared with normal hearing? We hypothesised that hearing loss would increase the risk of frailty in older adults aged 50 years or over.

## Methods

### Study Eligibility

This review focused on observational studies and was limited to papers published in English. We searched cross-sectional, cohort and case-control studies investigating the association between hearing loss and frailty. Studies were included if: (1) the subjects of the study were aged 50 years or above; (2) hearing loss was self-reported or measured by audiology tests; (3) the presence of frailty was ascertained through the use of a valid measure. Studies were excluded if: (1) the type or cause of individuals’ hearing loss was reported and the hearing loss may not be permanent, for example, it was conductive or caused by other health issues such as tumour or injury; (2) the study did not offer a clear description of hearing and frailty assessment.

### Search Strategy

We conducted a comprehensive literature search of Medline, PsychINFO, Embase, and the Cochrane Collaboration databases from 2000 to February 2021. This time limit was set because studies on frailty emerged prominently after 2000 according to previous review articles [4, 5, 24]. The following search terms and Boolean operators were used to source articles: (“Hearing impairment” OR “Hearing loss” OR deafness OR “hearing deficit”) AND (frailty OR frail OR morbidity OR mortality OR fall OR “physical function”). Additional studies were sought from article reference lists, review articles, conference abstracts, Google Scholar and Open Grey.

### Study Selection

Identified citations from the electronic and manual searches were screened for eligibility by RT and full text documents of potentially eligible articles were retrieved. These articles were further assessed for final eligibility by all authors.

### Data Extraction

Data extracted from the final articles included: authors, sample characteristics, study characteristics, measurement and criteria of hearing loss and frailty, and quantitative data for the purpose of meta-analysis. Authors were contacted when necessary if study information or data were not reported in the published articles.

### Quality Assessment and Risk of Bias

The quality of the articles were assessed using the Newcastle-Ottawa Scale (NOS)[25]. This scale was developed for use in cohort and case-control studies. The scale is adapted for cross-sectional studies, according to the work of Modesti et al. [26]. Articles were assessed according to eight items that are categorized into three domains, including selection, comparability and outcome. Articles were classified as good, fair or poor quality according to their score in each section. Good quality was defined as having three or four stars in the selection domain (maximum five), one or two stars in the comparability domain (maximum two), and two or three stars in the outcome domain (maximum three); Fair quality was defined as having two stars in the selection domain, one or two stars in the comparability domain, and two or three stars in the outcome domain; Poor quality was defined as having one or zero stars in the selection domain, or zero stars in the comparability domain, or one or zero stars in the outcome domain. Articles rated as having poor quality were excluded from the review. Quality assessment was conducted by RT and agreed by all authors.

### Statistical Analyses

Stata 16.1 software was used for the meta-analyses (StataCorp LLC, 2019) using the ‘metan’ command. We used the measure of risk and respective 95 % confidence interval of each individual study to calculate the overall summary estimate of risk for all studies. Results that were adjusted for confounders were used when available. In this review, we use the generic term ‘risk ratio’ to describe the odds ratio, relative risk or hazard ratio reported by the individual studies. Separate analyses were undertaken for different study designs (i.e., cross-sectional and cohort). A random effects model was chosen because of study heterogeneity. Most studies reported hearing and frailty status in a dichotomous manner. Some studies reported these in several categories according to their severity, such as normal, mild, moderate or severe for hearing loss, and normal, pre-frail or frail for frailty. For these studies, data of the most severely impaired category was used for the purposes of the meta-analyses.

### Heterogeneity and Sensitivity Analyses

Q and I^2^ tests were conducted to examine heterogeneity. Significant Q test results (P < 0.10) provides evidence of heterogeneity. I^2^ test was used to quantify heterogeneity. Heterogeneity with I^2^ values lower than 40 % is generally considered low, 41-60 % is considered medium, and I^2^ values over 60 % is considered high. Heterogeneity was then explored by conducting subgroup analyses and sensitivity analyses. Sensitivity analysis was conducted by repeating the meta-analysis while sequentially removing individual studies.

## Results

### Study Selection

**Fig. 1:**
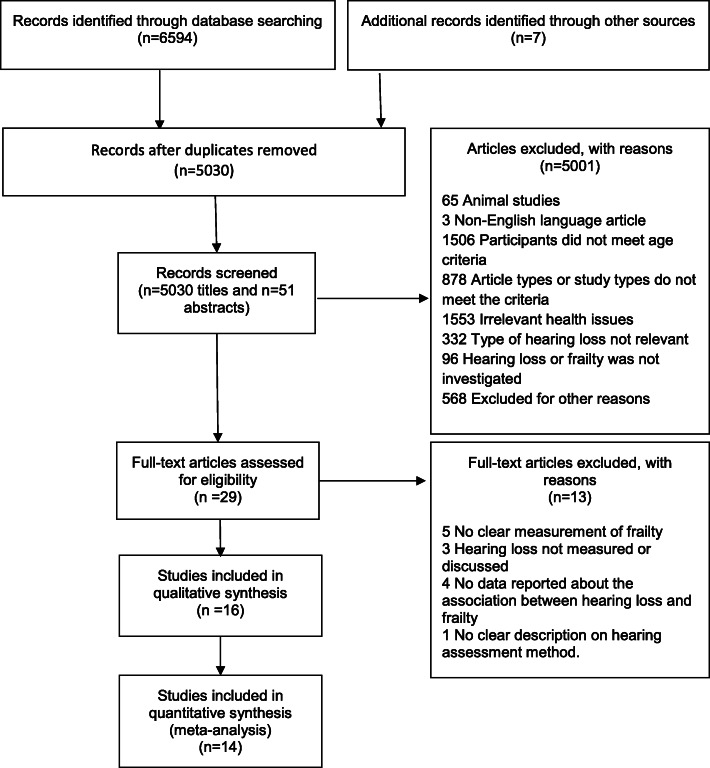
Flow chart showing results of the literature search

Figure 1 summarises the results of the systematic search of the literature. Five thousand and thirty (5030) articles were screened for eligibility, but only 16 fulfilled criteria for inclusion and these resulted in 13 cross-sectional and 5 longitudinal datasets (two studies reported both cross-sectional and longitudinal data [27, 28]). Among these, two studies [29, 30], and the longitudinal component of one study [27] did not include sufficient data for meta-analysis. Fourteen studies were included in the quantitative analysis with 12 sets of cross-sectional data (including 12,313 participants) and three sets of longitudinal data (including 3042 participants).No case-control studies met our inclusion criteria.

### Study Characteristics

Table 1 summarises the 16 included studies. Among them, nine studies identified hearing loss through simple self-reported questions [28, 31–33] (e.g. “Is your hearing excellent, very good, good, fair or poor?”), questionnaires [23, 34, 35], or subjective judgement of examiners [22, 27]. The other seven studies used validated audiology methods, including pure-tone audiometry [13, 36], whisper test [29, 30, 37, 38] and finger friction test [39], to measure hearing.

**Table 1:** Summary of included studies

Study Name	Study design	Participants	Hearing Assessment		Frailty Assessment		Measure of risk used in meta-analysis with 95 % CI	Quality Rating
			Method	Criteria	Method	Criteria		
Buttery et al., 2015 [34]	Cross-sectional	1843 (1184^a^) community-dwelling people aged between 65 to 79 in Germany.	*Self-reported* Multiple questions	Questions such as “Do you have problem on the telephone?“ “Do you have problem in groups of more than 4 people?“	Fried criteria	A participant without any of the 5 components was defined as nonfrail, 1 to 2 components as prefrail and 3 and more components as frail.	Relative risk ratio: 5.38 (2.17, 13.35)	Good
Cakmur, 2015 [29]	Cross-sectional	168 community-dwelling people aged above 65 in Turkey	*Audiology Assessment* Whisper test	A researcher stood 20–40 cm behind the individual, who had 1 ear closed, and the subject was asked to repeat something said by the researcher.	Fried criteria	A participant without any of the 5 components was defined as nonfrail, 1 to 2 components as prefrail and 3 and more components as frail.	Not included in meta-analysis	Good
Castellana et al., 2021 [36]	Cross-sectional	1929 (1156^a^) community-dwelling people aged above 65 in Italy	*Audiology Assessment* Audiometry	WHO standard: A PTA average at 0.5, 1, 2, and 4 kHz was calculated for the better hearing ear (disabling HI > 40 dB)	Fried criteria	A participant without any of the 5 components was defined as nonfrail, 1 to 2 components as prefrail and 3 and more components as frail.	Odds ratio: 1.48 (1.10, 2.01)	Good
Cheung et al., 2020 [27]	Cross-sectional and Cohort	306 (165^a^) people aged above 60 in Hong Kong	*Subjective* Validated tool (assessed by examiner)	Hearing item of the interRAI	Fried criteria	A participant without any of the 5 components was defined as nonfrail, 1 to 2 components as prefrail and 3 and more components as frail.	Cross-sectional Odds ratio: 2.83 (1.00, 8.01) Cohort component not include in meta-analysis	Good
Closs et al., 2016 [38]	Cross-sectional	521 (255^a^) community-dwelling people aged above 60 in Brazil	*Audiology Assessment* Whisper test	Whispered 33 cm behind the participant’s field of vision. Hearing impairment was defined as unable to answer the simple question.	Fried criteria	A participant without any of the 5 components was defined as nonfrail, 1 to 2 components as prefrail and 3 and more components as frail.	Odds ratio: 3.09 (1.73, 5.52)	Good
Doba et al., 2012 [35]	Cohort	407 community-dwelling people aged above70 in Japan.	*Self-reported* Multi-choice question	Hearing classified as none, slight, or obvious according to questionnaires.	CSHA Clinical Frailty Scale	Scored according to the scale. non-frail group scores from 1 to 3; frail group scores from 4 to 7.	Odds ratio: 2.186 (1.197, 3.995)	Good
Gu et al., 2019 [22]	Cross-sectional	4323 (2188^a^) community-dwelling people aged above 60 in China	*Subjective* Validated tool Assessed by examiner	Hearing: “clear-ly hearing” and “not clearly hearing or inaudible”, judged by examiners using voice test.	Fried criteria	A participant without any of the 5 components was defined as nonfrail, 1 to 2 components as prefrail and 3 and more components as frail.	Odds ratio: 1.30 (0.59, 2.87)	Good
Herr et al., 2018 [33]	Cross-sectional	1228 (867^a^) people aged 100 and above in Japan, France, Switzerland, Denmark, and Sweden	*Self-reported* Simple question	Major difficulties in hearing when talking to a single person in a quiet room or hearing a telephone conversation	Fried criteria	A participant without any of the 5 components was defined as nonfrail, 1 to 2 components as prefrail and 3 and more components as frail.	Odds ratio: 7.16 (3.24, 15.8)	Good
Kamil et al., 2014 [31]	Cross-sectional	2109 community-dwelling people aged 70 and above in the USA	*Self-reported* Simple question	Participants rated hearing as good, a little trouble or a lot of trouble. Defined as good to a little trouble hearing versus a lot of trouble hearing.	Fried criteria	A participant without any of the 5 components was defined as nonfrail, 1 to 2 components as prefrail and 3 and more components as frail.	Odds ratio: 1.68 (1.00, 2.82)	Good
Kamil et al., 2016 [13]	Cohort	2000 (1239^a^) community-dwelling people aged between 70 to 79 in the USA	*Audiology Assessment* Audiometry	WHO standard: A PTA average at 0.5, 1, 2, and 4 kHz was calculated for the better hearing ear (normal hearing ≤ 25 dB, mild HI = 26–40 dB, moderate-or-greater HI > 40 dB)	Physical frailty	A gait speed of less than 0.60 m/s; Inability to rise from a chair without using one’s arms. Positive for 1 test was considered frail, positive for both was considered severely frail.	Hazard ratio: 1.63 (1.26, 2.12)	Good
Liljas et al., 2017 [28]	Cross-sectional and Cohort	Community-dwelling people aged 60 and above in the UK. 2836 (1658^a^) participants in cross-sectional study; 1396 participants in cohort study.	*Self-reported* Validated question	Participants rated hearing as excellent, very good, good, fair, or poor. Defined as excellent to good hearing versus fair or poor hearing.	Fried criteria	A participant without any of the 5 components was defined as nonfrail, 1 to 2 components as prefrail and 3 and more components as frail.	Odds ratio: Cross-sectional: 1.52 (1.25, 1.86) Cohort: 1.32 (0.96, 1.81)	Good
Lorenzo-López et al., 2019 [30]	Cohort	749 community-dwelling people aged 65 and above in Spain.	*Audiology Assessment* Whispered-voice test	Whispered 0.6 m behind the participant’s field of vision. Hearing impairment was defined as unable to repeat back at least 3 out of a possible total of 6 letters/ numbers correctly.	Fried criteria	A participant without any of the 5 components was defined as nonfrail, 1 to 2 components as prefrail and 3 and more components as frail.	Not include in meta-analysis	Good
Mohd Hamidin et al., 2018 [23]	Cross-sectional	279 community-dwelling people aged 60 years and above in Malaysia	*Self-reported* questionnaire	Self-reported poor hearing	Fried criteria	A participant 2 or less components was defined as nonfrail, and 3 and more components as frail.	Odds ratio: 2.20 (0.91, 5.37)	Good
Naharci et al., 2019 [32]	Cross-sectional	484 community-dwelling people aged 60 and above in the USA.	*Self-reported* Single question	Participants rated hearing as excellent, very good, good, fair, or poor. Defined as excellent to good hearing versus fair or poor hearing.	Fried criteria	A participant without any of the 5 components was defined as nonfrail, 1 to 2 components as prefrail and 3 and more components as frail.	Odds ratio: 3.064 (1.422, 6.604)	Fair
Ng et at., 2014 [37]	Cross-sectional	1685 community-dwelling people aged 55 and above in Singapore.	*Audiology Assessment* Self-report and Standard whisper test	Standard whisper test	Fried criteria	A participant without any of the 5 components was defined as nonfrail, 1 to 2 components as prefrail and 3 and more components as frail.	Odds ratio: 2.34 (1.21, 4.52)	Good
Sable-Morita et al., 2018 [39]	Cross-sectional	283 outpatients with diabetes mellitus aged 65 and above in Japan.	*Audiology Assessment* Finger friction test	The examiner stood 30 cm behind the subject and made the noise 5 cm from each ear twice. Hearing impairment was defined as unable to hear the sound in both ears.	KCL score	This checklist consists of 7 domains: exercise/fall, instrumental activities of daily living, cognition, mood, malnutrition, oral function, and social activities of daily living. Frailty was defined as a total KCL score ≥ 8.	Odds ratio: 2.02 (1.085, 3.76)	Fair

Note: *95 % CI* 95 % confidence interval; *KCL* Kihon Checklist; *PTA* pure-tone audiometry; *CSHA* Canadian Study for Health and Aging

^a^Number of participants included in the systematic review. Different from total number reported by the study because only the most severely impaired category was used when several categories were reported

The studies used various methods to define frailty. Most (n = 13) used the Fried criteria [2] to define frailty status, but the criteria for each component were slightly modified, as described in Table 2. Kmail et al. (2016) [13] used two simple assessments to define physical frailty, Sable-Morita et al. [39] used the Kihon Checklist (KCL) score, and Doba et al. [35] used the Canadian Study for Health and Aging Clinical Frailty Scale (CSHA-CFS). The odds ratio of four studies [27, 33, 36, 38] used in the meta-analysis were calculated by us using demographic data reported by the studies. Another four studies [29, 30, 35, 37] reported only unadjusted statistics (i.e., not adjusted for covariates). All other eight studies [13, 22, 23, 28, 31, 32, 34, 39] were adjusted for age, gender, and other additional factors according to their study design.

**Table 2:** Summary of criteria used for Fried Phenotype components

Study	Fried Phenotype Components				
	Weight Loss	Gait Speed	Weakness	Exhaustion	Low Physical Activity
Buttery et al. [34]	Participants with a BMI of less than 23 were considered low weight.	The Timed Up and Go test is used. Participants taking 15 s or more were classified as having a low walking speed.	Isometric grip strength was measured using a hand-held dynamometer Low grip strength was determined using sex and BMI specific cut points specified by Fried.	Measured using a single item from the Medical Outcome SF-36. Participants answering ‘none’ or ‘little of the time’ to the question ‘How much of the time during the past four weeks did you have a lot of energy?’ were classified as having exhaustion.	Participants reporting performing no sports in the previous three months, and no physical activity on any day of the week requiring the person to start to sweat or get out of breath were classified as having low physical activity.
Cakmur [29]	Self-reported unintentional weight greater than 3 kg in the previous 3 months.	Slow gait speed was measured through the 6-meter walking speed test and was adjusted for gender and height.	Muscle weakness was measured with a hand dynamometer, and the average score for the dominant hand, adjusted for gender and BMI, was recorded.	Self-reported exhaustion was evaluated by asking: “Do you feel a lack of energy or fatigue or tiredness?”	The Independence in ADL index was used to evaluate low physical activity.
Castellana et al. [36]	Assessed by the Mini Nutritional Assessment, using a score threshold < 23.5.	Gait speed was evaluated using a 5-m walking test and rated slow if the time recorded was greater than or equal to the cut-off point of 0.6 m/s	The 5-repetitions sit-to-stand test measures the amount of time a patient takes to rise 5 times from a seated position without using his or her arms and was used as a proxy measure of weakness, > 15 s classified as weakness	Modified version of the Berg stool-stepping task	Participants reporting average level of physical activity during the past year, choosing from 6 response categories (from 0 to 5), < 2 classified as having low physical activity
Cheung et al. [27]	10 pounds or greater unintentional weight loss in the last year	Slow walking speed	Grip strength	Self-reported exhaustion	Physical Activity Scale for the Elderly
Closs et al. [38]	4.55 kg or greater unintentional weight loss in the last year	Walking a standard distance course of 4.6 m: ≥ 7 s for men height ≤ 1.73 m or women height ≤ 1.59 m; ≥ 6 s for men height > 1.73 m or women height > 1.59 m	Men: BMI ≤ 24 grip strength ≤ 29; BMI 24.1–26 grip strength ≤ 30; BMI 26.1–28 grip strength ≤ 30; BMI > 28 grip strength ≤ 32 Women: BMI ≤ 23 grip strength ≤ 17; BMI 23.1–26 grip strength ≤ 17.3; BMI 26.1–29 grip strength ≤ 18; BMI > 29 grip strength ≤ 21	Self-reported exhaustion, identified by two questions from the depression scale of CES-D	Minnesota Leisure Time Activity Questionnaire: < 383 kcal for men < 270 kcal for women
Gu et al. [22]	Greater than 4.55 kg or 5 % unintentional weight loss in the last year	Walking speed < 0.8 m/s over a distance of 4.57 m, or needing auxiliary walking equipment or human assistance while walking	Maximum value of grip strength <26 kg in male or <18 kg in female	Answer “Yes” when being asked “whether do you often feel fatigue?“	Defined as “low group” in the short form of international physical activity questionnaire
Herr et al. [33]	Self-reported weight loss of 5 kg during the past year; And/or self-reported weight loss of 3 kg during the past 3 months; And/or body mass index ≤ 18.5 kg/m2	Self-reported slow walking speed And/or difficulty walking up a flight of stairs And/or bedridden or unable to transfer from bed to chair without help	Self-reported difficulty carrying a bag weighting 5 kg	Self-reported fatigue (when moving, resting,or all the time)	No regular exercise or outdoor activity (self-reported); And/or bedridden or unable to transfer from bed to chair without help
Kamil et al. (2014) [31]	5 % or greater unintentional weight loss in the last year or body mass index less than 18.5 kg/m.	20-foot gait speed in the lowest sex-adjusted quintile.	Self-reported weakness (some or much difficulty lifting or carrying an object as heavy as 10 pounds or unable to do).	Self-reported exhaustion (some or much difficulty walking from one room to another or unable to do).	Self-reported low physical activity (participant report of being less active than individuals of the same age).
Liljas et al. [28]	Weight loss was defined as loss of 10 % or more of body weight in the last 4 years or a current BMI of less than 18.5 kg/m2.	Slow walking speed was measured as the mean time of two measurements taken to complete an 8-foot walk at usual pace.	Assessed using a dynamometer with the maximum handgrip strength measure out of three attempts on each side used for analysis. Weak grip was classified as being in the lowest quintile of the sex- and BMI-adjusted distribution.	Self-reported exhaustion identified by two questions (items 7 and 20) from the CES-D scale. Exhaustion was defined as a positive response to either of the two statements from the CES-D (items 7 and 20).	Based on three questions about the frequency with which participants undertook vigorous, moderate, and mild exercise.
Lorenzo-López et al. [30]	Unintentional weight loss greater than 4.5 kg in previous year.	The walking time (in seconds) over a distance of 4.57 m, adjusting for gender and height.	Muscle weakness was measured with a hand dynamometer in the dominant hand, results adjusted for gender and BMI.	Self-reported exhaustion, identified by two questions (items 7 and 20) from the CES-D scale.	Measured by the weighted score of kilocalories expended per week, calculated based on the Minnesota Leisure Time Activity Questionnaire, based on each participant’s report, and adjusting for gender.
Mohd Hamidin et al. [23]	Greater than 4.5 kg or 5 % unintentional weight loss in the last year	Walking a standard distance course of 4.6 m: ≥ 7 s for men height ≤ 1.73 m or women height ≤ 1.59 m; ≥ 6 s for men height > 1.73 m or women height > 1.59 m	Participants who cannot carry out the muscle strength test, or who recorded less than 18.0 kg of grip strength for male and 12.5 kg for female, were classified as positive for grip strength criterion.	Self-reported exhaustion was defined as a positive response to either of the two statements from the CES-D (items 7 and 20).	Assessed according to self-report of frequency, duration and intensity of usual activities based on the Rapid Assessment of Physical Activity questionnaires
Naharci et al. [32]	Unintentional loss of 10 or more pounds in the last six months.	Participants were asked to walk without assistance for 25 feet, and the time was measured for the intermediate 15 feet. Cut-offs stratified by gender and height were used, and slow walking speed was defined according to Fried criteria.	Grip strength was measured using dynamometers on the dominant hand. The average of two attempts was used, and the cut-offs were stratified by gender and BMI quartiles. The lowest quartile in each gender group was considered weak for the frailty criteria.	Self-reported exhaustion identified by two questions (items 7 and 20) from the CES-D scale. Exhaustion was defined as a positive response to either of the two statements from the CES-D (items 7 and 20).	Participants were asked how often they engaged in vigorous, moderate and mild activities [17], and were classified as having low physical activity if they answered ‘hardly ever or never’ or ‘one to three times a month’ to all three of the questions.
Ng et at [37]	BMI of less than 18.5 kg/m2 and/or unintentional weight loss of 10 pounds (4.5 kg) or more in the past 6 months.	Using the 6-meter fast gait speed test, using the average of 2 measurements, and the lowest quintile values stratified for gender and height to classify participants as slow.	Leg muscle strength was determined using dominant knee extension, using the average value from 3 trials in kilograms, standardized on gender and BMI strata. Participants with knee extension strengths in the lowest quintiles were classified as weak.	Measured with 3 questions on vitality domain in the Medical Outcomes Study SF-12: “Did you feel worn out?” “Did you feel tired?” “Did you have a lot of energy?” with total summed scores ranging from 3 to 15, and a higher score indicating more energy. A score of less than 10 was used to denote exhaustion.	Physical activities were assessed based on self-reported time (in hours) spent doing light, moderate, and vigorous activities on weekdays and the weekend. The total amount of time spent on performing moderate and vigorous activities per week and activity time below the gender-specific lowest quintile was used to denote frailty.

*Note: BMI *Body mass index*; CES-D *Center for Epidemiologic Studies Depression*; ADL *Activities of Daily Living

Follow-up periods for the five longitudinal studies varied, with 10 years of follow up reported by Kamil et al. (2016) [13], five years by Doba et al. [35] and Cheung et al. [27], four years by Liljas et al. [28] and one year by Lorenzo-López et al. [30].

Three studies were conducted in the USA [13, 31, 32], six studies based in Asia [22, 23, 27, 35, 37, 39], four in Europe [28, 30, 34, 36], one each in Turkey [29] and Brazil [38], and one study [33] was a multinational study involving five countries. Most of the participants were community dwelling aged people, while Sable-Morita et al. [39] recruited participants from a diabetes mellitus outpatient clinic, and Cheung et al. [27] recruited participants through aged-care services. Herr et al. [33] did not specify the recruitment setting.

Fourteen studies were included in the meta-analysis. Among them, seven [22, 23, 28, 31, 32, 35, 37, 39] reported odds ratios, while Kamil et al. (2016) [13] reported hazard ratio and Buttery et al. [34] reported relative risk ratio. We calculated the odds ratios of four studies [27, 33, 36, 38]. Two studies were not suitable for meta-analysis. Cakmur [29] completed a cross-sectional study of 168 older participants in rural Turkey, aiming to investigating the prevalence of frailty in this area and its correlates. Hearing loss was reported to have a statistically significant association with frailty, but the specific data were not described. Lorenzo-López et al. [30] reported the results of a longitudinal study involving 749 community-dwelling older adults in Spain and followed their frailty status for one year. The frailty status transitions (progressed, regressed, no change or death) and their associated factors were reported, but these data could not be included in the meta-analyses because it did not report the risk of incident frailty. In this study, hearing impairment at baseline was associated with higher risk of experiencing worsening of frailty over time. Cheung et al. [27]reported that hearing impairment is associated with worse frailty status transition over a 5-year period. These longitudinal data are not included in the meta-analysis, but we calculated the odds ratio for the cross-sectional data and included this in the meta-analysis.

### Risk of Bias Within Studies

Study quality is summarised in eTable 2. According to the Newcastle-Ottawa Scale (NOS) [25] the quality of the studies included in the review were good or fair. The quality of two studies were considered as fair. No cross-sectional studies reported characteristics of the non-responders, but two [23, 33] reported the response rate. One possible reason for this could be that the data used in most cross-sectional studies were from previous studies and the samples were restricted to participants with information available to address the aims of the studies. Thus, non-response rate was not available. Participants of Sable-Morita et al. [39] were outpatients with diabetes recruited from a single clinic, which limits the generalisability of the findings. The sample size of this study, and of Naharci et al. [32], Cheung et al. [27], Closs et al. [38] and Mohd Hamidin et al. [23] was relatively modest, with 283, 484, 165, 255 and 279 participants, respectively. Among the longitudinal studies, Lorenzo-López et al. [30] reported that 28.3 % of participants were lost to follow-up. Liljas et al. [28] used prospective data from a national study and only participants with complete data were included in the study. In Doba et al. [35], assessment measures were self-rating. The forms were later reviewed by trained nurses, but the validity of the assessment of hearing loss seemed less certain than that of a structured interview, test or health record. Kamil et al. (2016) [13] reported 35 participants were frail or severely frail, but these people were not excluded from follow-up, so that prevalent cases may have contaminated the sample.

### Meta-analysis of the primary outcome - hearing loss and frailty

**Fig. 2:**
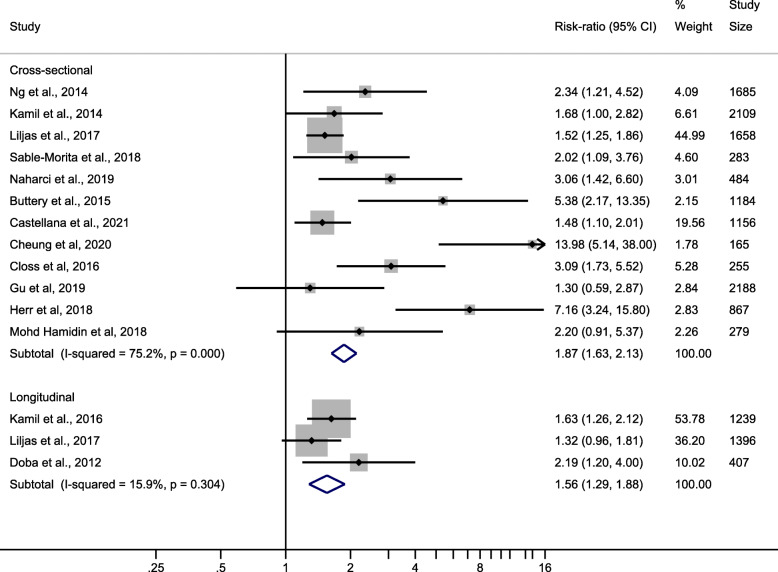
Forrest plot showing overall risk ratio of cross-sectional studies and longitudinal studies

The overall association between hearing loss and frailty for cross-sectional studies included in this meta-analysis was associated with a RR of 1.87 (95 %CI 1.63–2.13). For longitudinal studies the RR was 1.56 (95 %CI 1.29–1.88) – see Fig. 2.

### Risk of bias across studies

There was high (I^2^ = 75.2 %) and low (I^2^ = 15.9 %) heterogeneity present in the meta-analysis of cross-sectional and longitudinal studies, respectively. The funnel plots indicated that publication bias may have been present and that positive studies may have been more likely to appear in print (figure not shown).

### Sensitivity Analyses

Sensitivity analyses were conducted for cross-sectional studies to determine the impact of individual reports on the outcome of the meta-analysis. The meta-analysis was repeated by sequentially removing individual studies – the results are depicted in eFigure 1. These were not performed for longitudinal studies due to the small number of publications available. No single study had a significant effect on the overall effect, although removal of Liljas et al. [28] did result in a larger summary effect estimate (RR 2.21, 95 %CI 1.84–2.64). The heterogeneity remained high, whichever study was removed.

### Subgroup analyses

Subgroup analyses were conducted for cross-sectional studies according to the methods used to assess hearing, the sample size and the quality of studies. Longitudinal studies were not included in these additional analyses because of the limited number of studies.

#### Hearing assessment method

**Fig. 3:**
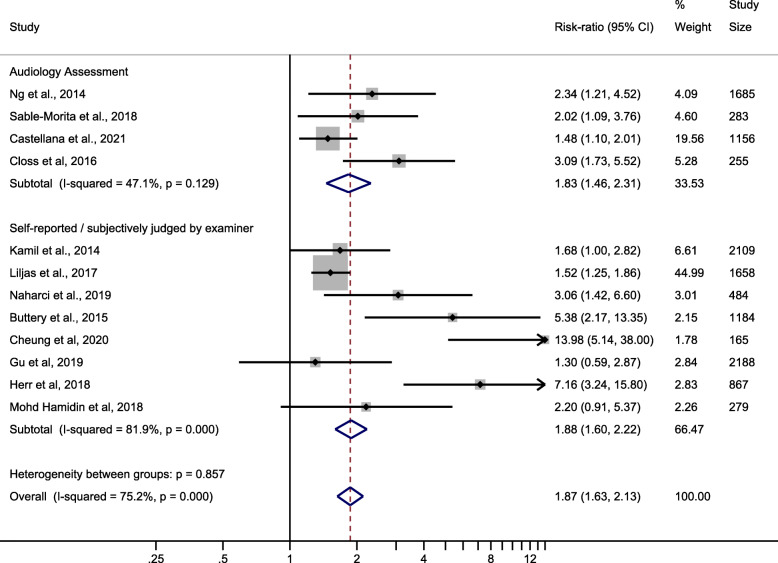
Subgroup analyses of cross-sectional studies according to the methods used to assess hearing ability

Among cross-sectional studies, Castellana et al. [36], Closs et al. [38], Ng et al. [37] and Sable-Morita et al. [39] used validated audiology assessment method to identify hearing loss, while in the rest eight studies hearing ability were either self-reported [23, 28, 31–34] or subjectively judged by examiners [22, 27]. When separately analysed (Fig. 3), both groups had similar pooled effect size, but the group using an audiology assessment method had a much smaller heterogeneity when compared with the other group (RR 1.83, 95 %CI 1.46–2.31, I^2^ = 47.1 % vs. RR 1.88, 95 %CI 1.60–2.22, I^2^ = 81.9 %).

#### Sample size

The association between hearing loss and frailty remained significant regardless of study size although heterogeneity did vary across the three sample size categories (eFigure 2). The group of study with more than 2000 participants had the smallest heterogeneity. The effect size of this association seemed more pronounced for the studies with participants less than 1000. However, only a small number of studies were available for each category.

#### Quality of studies

Estimates of study quality did not seem to affect the results of the meta-analysis (eFigure 3), although heterogeneity was higher in those studies rated as ‘good’ quality compared with ‘fair’ quality (I^2^ = 78.9 % vs. I^2^ = 0 %). The summary effect was also higher in the ‘fair’ quality studies, but it was associated with a less precise effect estimate (RR 2.38, 95 %CI 1.47–3.86 vs RR 1.83, 95 %CI 1.59–2.10).

## Discussion

The aim of this systematic review and meta-analysis was to determine if there is an association between hearing loss and frailty by reviewing currently available studies. We found that the risk of frailty is greater among older people with than without hearing loss, regardless of study design (cross-sectional or cohort), method of assessment of hearing and frailty, sample size or study quality.

Apart from the positive association between hearing loss and increased risk of frailty, other findings merit mention. Kamil et al. (2014) [31] showed that self-reported hearing loss was associated with frailty in women but not men. However, their subsequent longitudinal study in 2016 [13] using objective hearing assessment found increased risk for both men and women with moderate or greater hearing loss. They also reported that the use of hearing aids was not significantly associated with decreased frailty risk [31]. Sable-Morita et al. [39] reported higher risk of frailty in hearing impaired older adults with diabetes. They also indicated that previous studies suggested that people with diabetes may have higher prevalence of hearing loss than those without, although the physiological mechanisms supporting the purported association remain unclear. Naharci et al. [32] evaluated the association between self-reported hearing loss and frailty among four ethnic groups and observed an increase in risk only among Afro-Caribbeans, but not in African Americans, Hispanics or European Americans. Their sampling and assessment strategies (e.g., possible unbalanced distribution of severe hearing loss in the samples) could explain their results.

There are a number of strengths and limitations worth considering in this review. The possible publication bias suggests that the effect of the association between hearing loss and frailty may have been overestimated. Limiting our literature search to English language may also raise questions regarding the generalisability of the findings. We were only able to include 14 publications with 15 separate datasets (total number of participants = 13,959) in the meta-analyses, thereby limiting the power and generalizability of the study. Nonetheless, most studies included in this review had good methodological quality and our sensitivity and subgroup analyses suggest that the observed associations are most likely robust.

Heterogeneity, especially clinical heterogeneity, needs to be considered. Hearing ability in most studies were self-reported or measured by simple screening test such as whisper test or finger friction test. The self-reported degree of hearing impairment tends to be underestimated by middle-aged to older adults [40], so that false negatives may have contaminated the samples. Only two studies used pure-tone audiometry test, which provides an accurate result of individual’s hearing thresholds. Also, most of the studies we reviewed did not report information about the degree of hearing loss. The results would have been more informative if participants within the same degree of hearing were analysed separately, as the effect of hearing loss on the risk of frailty may be ‘dose-dependent’. Kamil et al. (2016) [13] is one of the two studies that used pure-tone audiometry, reporting a 11 % increased risk of frailty with per 10 dB increase in hearing thresholds. Future studies should consider excluding participants with hearing loss caused by reasons other than presbycusis and distinguishing participants whose hearing loss has/has not been treated.

Likewise, the heterogeneity associated with the assessment of frailty raises doubts about how best to interpret these results. Most of the studies (*n* = 15)[13, 35] that we included in these analyses focused on physical frailty only, while Sable-Morita et al. [39] used the KCL, and considered frailty included not only physical, but also social and psychological aspects. This checklist assesses seven areas: exercise/fall, instrumental activities of daily living, cognition, mood, malnutrition, oral function, and social activities of daily living. It is similar to the Fried phenotype, albeit more inclusive. Thirteen studies used slightly modified Fried criteria. In addition, Naharci et al. [32] noted a higher proportion of exhaustion among people with than without self-reported hearing loss. There may be merit in exploring the association between hearing loss and each frailty domain in greater detail.

Eleven out of 16 articles reviewed used available data from existing population studies. These large studies collect data from multi-disciplines and were not specifically designed for hearing and frailty analysis. This could account for the limitations in study design in most of the articles we reviewed. Moreover, most studies had a cross-sectional design, so that the direction of the observed associations was not clear. Five articles reported longitudinal results, but follow-up periods varied from 1 year to 10 years.

Currently there is insufficient evidence to conclude that a causal relationship between hearing loss and frailty exists. More research is needed to investigate the plausibility of such hypotheses. It is important to further confirm and investigate this relationship. If hearing loss is found to be a marker of frailty, then health care providers should consider medical review for frail or pre-frail status when hearing loss is present. If hearing loss contributes significantly to frailty, then appropriate management may delay frailty and death in later life. Either way, considering the high prevalence of hearing loss among aged adults and the relatively straightforward management of this, regular assessments of older adults at risk of hearing loss and the proper management of hearing impairment could improve the quality of life of older adults and, potentially, lower costs of healthcare and social support systems by delaying the onset of frailty.

## Conclusions

Our review and meta-analysis of observational studies suggest that hearing loss is associated with higher prevalence and risk of frailty in later life. Whether this relationship is causal remains to be determined.

## Supplementary Information

**Additional file 1.**

## Data Availability

All data generated or analysed during this study are included in this published article and its supplementary information files.

## Abbreviations

ARHL: Age-related hearing loss
CSHA-CFS: Canadian Study for Health and Aging Clinical Frailty Scale
KCL: Kihon Checklist
NOS: Newcastle-Ottawa Scale
RR: Risk ratio
