# Supplementary material for: Association between hearing loss and frailty: a systematic review and meta-analysis
Source: BMC Geriatr. 2021 May 25;21:333. doi: 10.1186/s12877-021-02274-y (PMC8147347; doi:10.1186/s12877-021-02274-y)
Supplement: Supplementary file 1 — Additional file 1. [file 12877_2021_2274_MOESM1_ESM.pdf]

Supplemental Material

eTable 1. PRISMA Checklist

eTable 2. Quality assessment of the reviewed studies

eFigure 1. Graph showing overall risk ratio and heterogeneity with each study removed

eFigure 2. Subgroup analyses according to the sample size of studies

eFigure 3. Subgroup analyses according to quality of studies

eTable 1. PRISMA Checklist

Section/topic	#	Checklist item	Reported on Page #
**TITLE**			
Title	1	Identify the report as a systematic review, meta-analysis, or both.	1, title
**ABSTRACT**			
Structured summary	2	Provide a structured summary including, as applicable: background; objectives; data sources; study eligibility criteria, participants, and interventions; study appraisal and synthesis methods; results; limitations; conclusions and implications of key findings; systematic review registration number.	2, abstract
**INTRODUCTION**			
Rationale	3	Describe the rationale for the review in the context of what is already known.	3, introduction
Objectives	4	Provide an explicit statement of questions being addressed with reference to participants, interventions, comparisons, outcomes, and study design (PICOS).	4, introduction
**METHODS**			
Protocol and registration	5	Indicate if a review protocol exists, if and where it can be accessed (e.g., Web address), and, if available, provide registration information including registration number.	No
Eligibility criteria	6	Specify study characteristics (e.g., PICOS, length of follow-up) and report characteristics (e.g., years considered, language, publication status) used as criteria for eligibility, giving rationale.	4-5, Study Eligibility
Information sources	7	Describe all information sources (e.g., databases with dates of coverage, contact with study authors to identify additional studies) in the search and date last searched.	5, search strategy
Search	8	Present full electronic search strategy for at least one database, including any limits used, such that it could be repeated.	5, search strategy
Study selection	9	State the process for selecting studies (i.e., screening, eligibility, included in systematic review, and, if applicable, included in the meta-analysis).	5, study selection
Data collection process	10	Describe method of data extraction from reports (e.g., piloted forms, independently, in duplicate) and any processes for obtaining and confirming data from investigators.	6, data extraction
Data items	11	List and define all variables for which data were sought (e.g., PICOS, funding sources) and any assumptions and simplifications made.	6, data extraction

Risk of bias in individual studies	12	Describe methods used for assessing risk of bias of individual studies (including specification of whether this was done at the study or outcome level), and how this information is to be used in any data synthesis.	6, quality assessment and risk of bias
Summary measures	13	State the principal summary measures (e.g., risk ratio, difference in means).	6-7, statistical analyses
Synthesis of results	14	Describe the methods of handling data and combining results of studies, if done, including measures of consistency (e.g., I^2) for each meta-analysis.	6-7, statistical analyses & heterogeneity and sensitivity analyses
Risk of bias across studies	15	Specify any assessment of risk of bias that may affect the cumulative evidence (e.g., publication bias, selective reporting within studies).	6, quality assessment and risk of bias
Additional analyses	16	Describe methods of additional analyses (e.g., sensitivity or subgroup analyses, meta-regression), if done, indicating which were pre-specified.	7, heterogeneity and sensitivity analyses
**RESULTS**			
Study selection	17	Give numbers of studies screened, assessed for eligibility, and included in the review, with reasons for exclusions at each stage, ideally with a flow diagram.	7, study selection
Study characteristics	18	For each study, present characteristics for which data were extracted (e.g., study size, PICOS, follow-up period) and provide the citations.	8-9, study characteristics; Table 1
Risk of bias within studies	19	Present data on risk of bias of each study and, if available, any outcome level assessment (see item 12).	9-10, risk of bias within studies; Table 1 & supplemental material etable 2
Results of individual studies	20	For all outcomes considered (benefits or harms), present, for each study: (a) simple summary data for each intervention group (b) effect estimates and confidence intervals, ideally with a forest plot.	10-12, figure 2, figure 3, supplemental material efigure 1, efigure 2, efigure 3

Synthesis of results	21	Present results of each meta-analysis done, including confidence intervals and measures of consistency.	10-12, figure 2, figure 3, supplemental material efigure 1, efigure 2, efigure 3
Risk of bias across studies	22	Present results of any assessment of risk of bias across studies (see Item 15).	10-11, risk of bias across studies
Additional analysis	23	Give results of additional analyses, if done (e.g., sensitivity or subgroup analyses, meta-regression [see Item 16]).	10-12, figure 3, supplemental material efigure 1, efigure 2, efigure 3
**DISCUSSION**			
Summary of evidence	24	Summarize the main findings including the strength of evidence for each main outcome; consider their relevance to key groups (e.g., healthcare providers, users, and policy makers).	12,14, discussion
Limitations	25	Discuss limitations at study and outcome level (e.g., risk of bias), and at review-level (e.g., incomplete retrieval of identified research, reporting bias).	13-14, discussion
Conclusions	26	Provide a general interpretation of the results in the context of other evidence, and implications for future research.	15, conclusion
**FUNDING**			
Funding	27	Describe sources of funding for the systematic review and other support (e.g., supply of data); role of funders for the systematic review.	15, funding

From: Moher D, Liberati A, Tetzlaff J, Altman DG, The PRISMA Group (2009). Preferred Reporting Items for Systematic Reviews and Meta-Analyses: The PRISMA Statement. PLoS Med 6(7): e1000097. doi:10.1371/journal.pmed1000097

eTable 2. Quality assessment of the reviewed studies

		Buttery et al (1)	Cakmur (2)	Castellana et al (3)	Cheung et al (4) Cross-sectional	Closs et al (5)	Kamil et al (2014) (6)	Liljas et al (7) Cross-sectional	Naharci et al (8)	Ng et at (9)	Sable-Morita et al (10)	Gu et al, (11)
Selection (Maximum 5)	Representativeness of the exposed cohort[a]	1	1	1	1	1	1	1	1	1	0	1
	Selection of the non-exposed cohort[a] / Sufficient simple size[b]	1	1	1	0	0	1	1	0	1	0	1
	Ascertainment of exposure[a b]	1	2	2	2	2	1	2	1	2	2	1
	Demonstration that outcome of interest was not present at start of study[a]	N/A	N/A	N/A	N/A	N/A	N/A	N/A	N/A	N/A	N/A	N/A
	Description of non-respondents[b]	0	0	0	0	0	0	0	0	0	0	0
Comparability (Maximum 2)	Comparability of cohorts on the basis of the design or analysis controlled for confounders[a b]	2	1	1	1	1	2	2	2	1	2	2
Outcome (Maximum 3)	Assessment of outcome[a b c]	2	2	2	2	2	2	2	2	2	2	2
	Was follow-up long enough for outcomes to occur[a]	N/A	N/A	N/A	N/A	N/A	N/A	N/A	N/A	N/A	N/A	N/A
	Adequacy of follow-up of cohorts[a]	N/A	N/A	N/A	N/A	N/A	N/A	N/A	N/A	N/A	N/A	N/A
	Statistical test[b]	1	1	1	1	1	1	1	1	1	1	1
Overall quality		Good	Good	Good	Good	Good	Good	Good	Fair	Good	Fair	Good

		Herr et al (12)	Mohd Hamidin et al (13)	Cheung et al (4) Longitudinal	Doba N et al (14)	Kamil et al 2016 (15)	Liljas et al (7) Longitudinal	Lorenzo-López et al (16)
Selection (Maximum 5)	Representativeness of the exposed cohort[a]	1	1	1	1	1	1	1
	Selection of the non-exposed cohort[a] / Sufficient simple size[b]	1	1	1	1	1	1	1
	Ascertainment of exposure[a b]	1	1	2	0	2	2	1
	Demonstration that outcome of interest was not present at start of study[a]	N/A	N/A	1	1	0	1	1
	Description of non-respondents[b]	1	1	N/A	N/A	N/A	N/A	N/A
Comparability (Maximum 2)	Comparability of cohorts on the basis of the design or analysis controlled for confounders[a b]	1	2	1	1	2	2	1
Outcome (Maximum 3)	Assessment of outcome[a b c]	2	2	1	1	1	1	1
	Was follow-up long enough for outcomes to occur[a]	N/A	N/A	1	1	1	1	1
	Adequacy of follow-up of cohorts[a]	N/A	N/A	1	1	1	0	0
	Statistical test[b]	1	1	N/A	N/A	N/A	N/A	N/A
Overall quality		Good	Good	Good	Good	Good	Good	Good

Note: [a] Items for cohort study. [b] Items adapted for cross-sectional study, according to the work of Modesti et al (17). [c] Maximum 1 point for cohort study, 2 for cross-sectional study. Good quality: 3 or 4 stars in selection domain AND 1 or 2 stars in comparability domain AND 2 or 3 stars in outcome domain; Fair quality: 2 stars in selection domain AND 1 or 2 stars in comparability domain AND 2 or 3 stars in outcome domain; Poor quality: 0 or 1 star in selection domain OR 0 stars in comparability domain OR 0 or 1 stars in outcome domain.

eFigure 1. Graph showing overall risk ratio and heterogeneity with each study removed

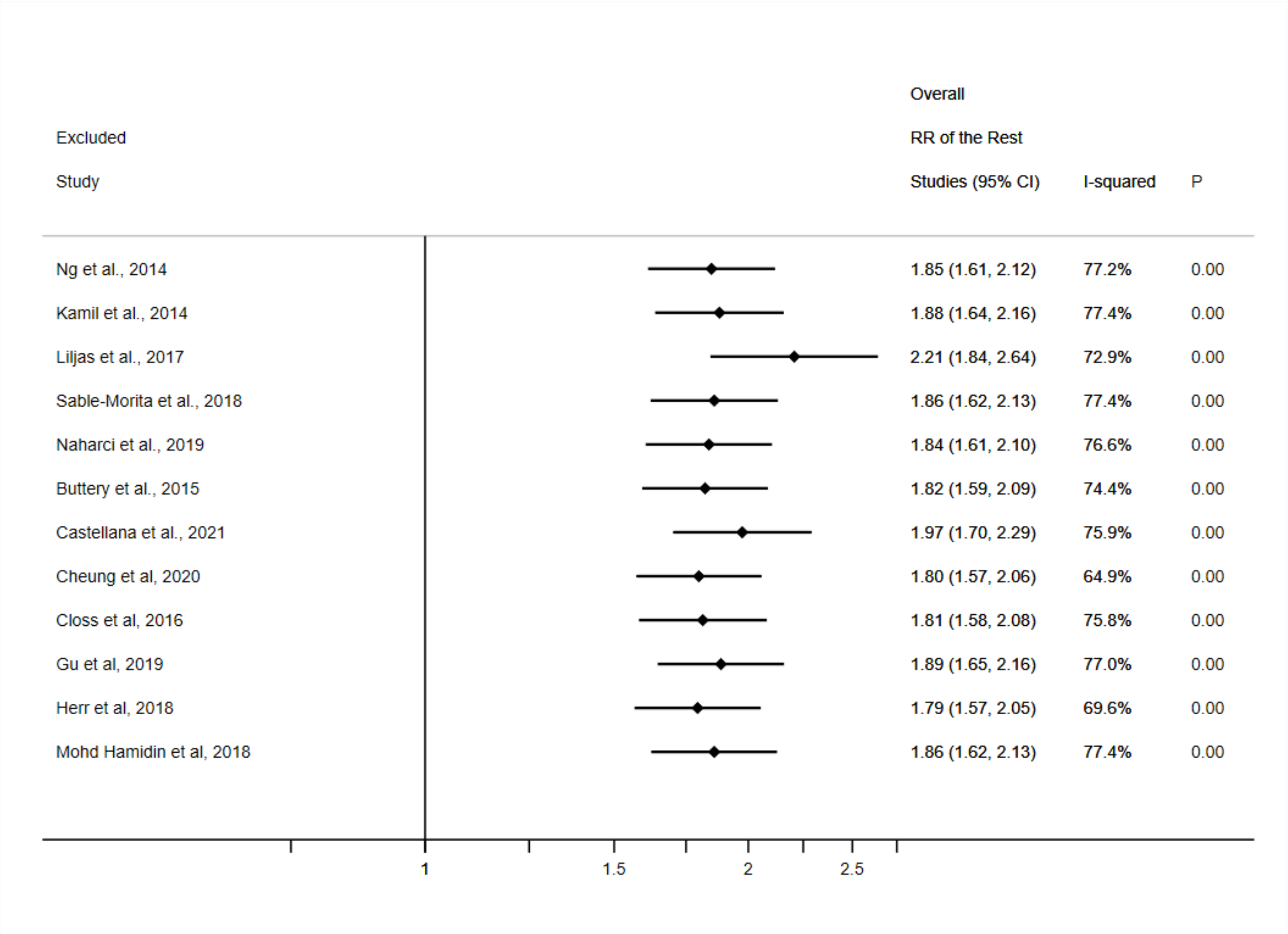

eFigure 2. Subgroup analyses according to the sample size of studies

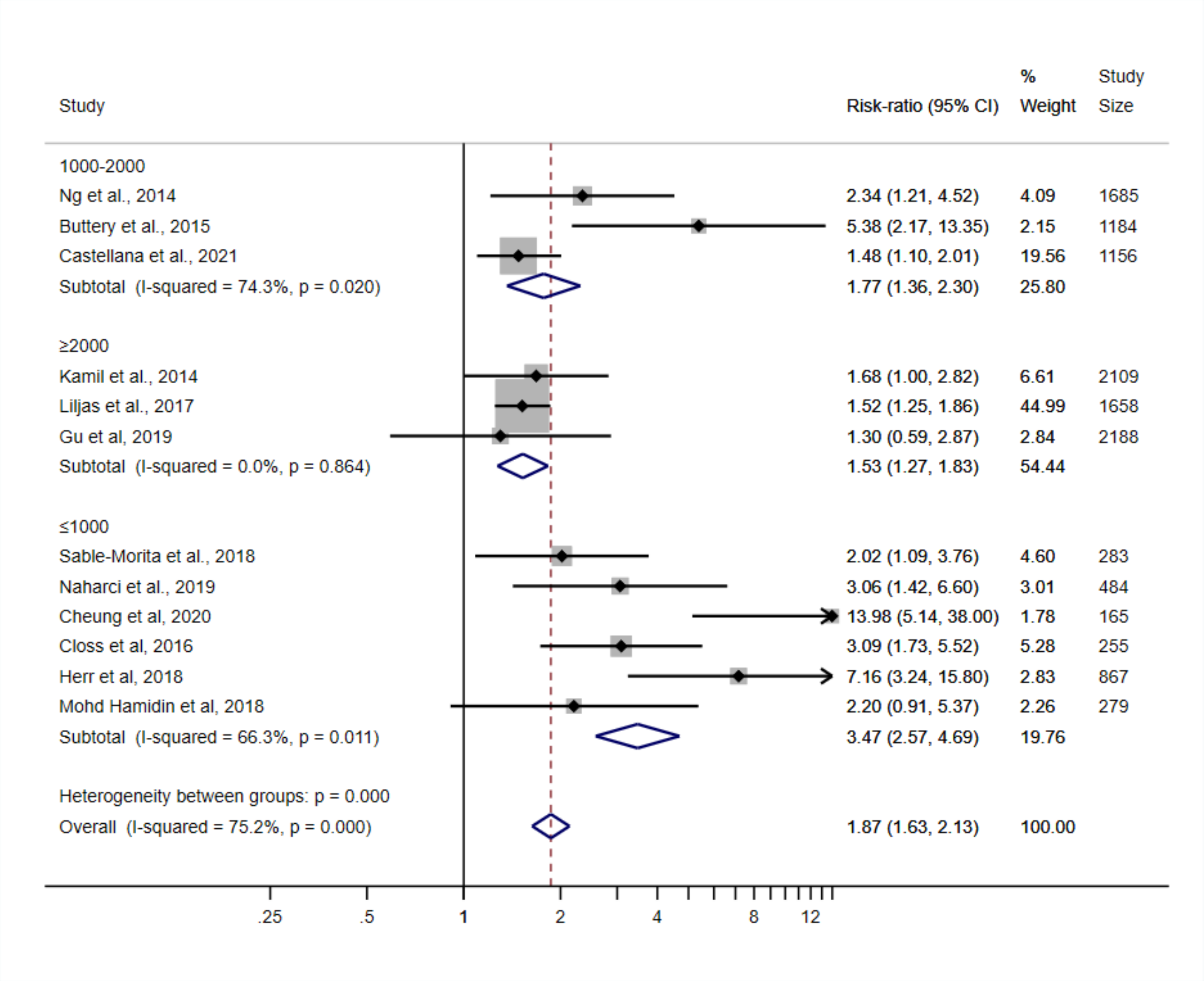

eFigure 3. Subgroup analyses according to quality of studies

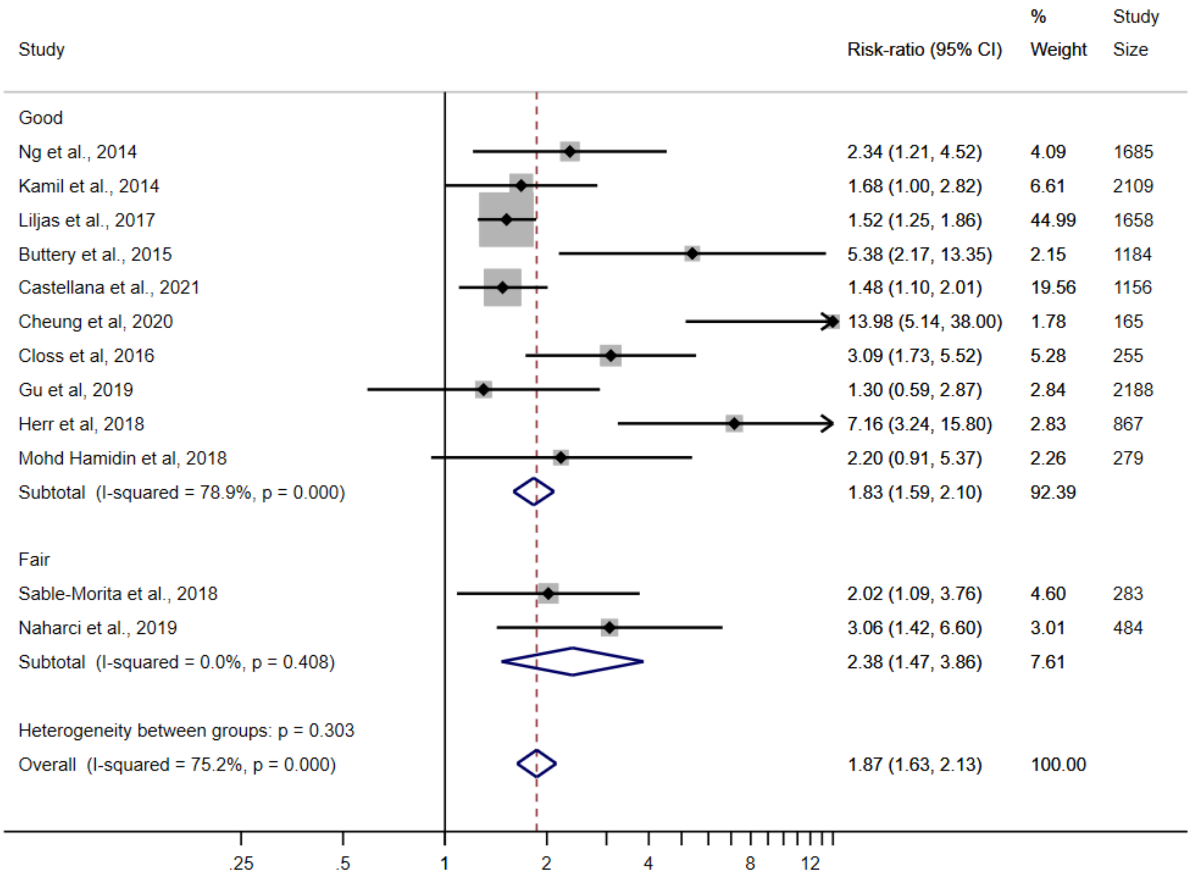

1. Buttery AK, Busch MA, Gaertner B, Scheidt-Nave C, Fuchs J. Prevalence and correlates of frailty among older adults: Findings from the german health interview and examination survey. *BMC Geriatrics.* 2015;15(1):22. doi:10.1186/s12877-015-0022-3.

2. Cakmur H. Frailty among elderly adults in a rural area of turkey. *Med Sci Monit.* 2015;21:1232-1242. doi:10.12659/MSM.893400.

3. Castellana F, Lampignano L, Bortone I, et al. Physical frailty, multimorbidity, and all-cause mortality in an older population from southern italy: Results from the salus in apulia study. *J Am Med Dir Assoc.* 2021. doi:10.1016/j.jamda.2020.12.026.

4. Cheung DSK, Kwan RYC, Wong ASW, et al. Factors associated with improving or worsening the state of frailty: A secondary data analysis of a 5-year longitudinal study. *J Nurs Scholarsh.* 2020;52(5):515-526. doi:10.1111/jnu.12588.

5. Closs VE, Ziegelmann PK, Gomes I, Schwanke CHA. Frailty and geriatric syndromes in elderly assisted in primary health care. *Acta Scientiarum Health Sciences.* 2016;38(1). doi:10.4025/actascihealthsci.v38i1.26327.

6. Kamil RJ, Li L, Lin FR. Association between hearing impairment and frailty in older adults. *J AM GERIATR SOC.* 2014;62(6):1186-1188. doi:10.1111/jgs.12860.

7. Liljas AEM, Carvalho LA, Papachristou E, et al. Self-reported hearing impairment and incident frailty in english community-dwelling older adults: A 4-year follow-up study. *J AM GERIATR SOC.* 2017;65(5):958-965. doi:10.1111/jgs.14687.

8. Naharci MI, Engstrom G, Keintz C, Danesh A, Tappen R, Ouslander JG. Self-reported hearing loss is associated with frailty among afro-caribbeans. *W INDIAN MED J.* 2019;68(1):29-34. doi:10.7727/wimj.2016.174.

9. Ng TP, Feng L, Nyunt MS, Larbi A, Yap KB. Frailty in older persons: Multisystem risk factors and the frailty risk index (fri). *J AM MED DIR ASSOC.* 2014;15(9):635-642. doi:10.1016/j.jamda.2014.03.008.

10. Sable-Morita S, Sugiura S, Uchida Y, Tanikawa T, Tokuda H, Arai H. Relationship between hearing impairment and frailty in older patients with diabetes mellitus. *European Geriatric Medicine.* 2017;8 (Supplement 1):S109-S110. doi:10.4172/2576-3946.1000114.

11. Gu J, Chen H, Gu X, et al. Frailty and associated risk factors in elderly people with health examination in rural areas of china. *Iran J Public Health.* 2019;48(9):1663-1670.

12. Herr M, Jeune B, Fors S, et al. Frailty and associated factors among centenarians in the 5-coop countries. *Gerontology.* 2018;64(6):521-531. doi:10.1159/000489955.

13. Mohd Hamidin FA, Adznam SN, Ibrahim Z, Chan YM, Abdul Aziz NH. Prevalence of frailty syndrome and its associated factors among community-dwelling elderly in east coast of peninsular malaysia. *SAGE Open Med.* 2018;6:2050312118775581. doi:10.1177/2050312118775581.

14. Doba N, Tokuda Y, Goldstein NE, Kushiro T, Hinohara S. A pilot trial to predict frailty syndrome: The japanese health research volunteer study. *Experimental Gerontology.* 2012;47(8):638-643. doi:10.1016/j.exger.2012.05.016.

15. Kamil RJ, Betz J, Powers BB, et al. Association of hearing impairment with incident frailty and falls in older adults. *J Aging Health.* 2016;28(4):644-660. doi:10.1177/0898264315608730.

16. Lorenzo-López L, López-López R, Maseda A, Buján A, Rodríguez-Villamil JL, Millán-Calenti JC. Changes in frailty status in a community-dwelling cohort of older adults: The verisaúde study. *Maturitas.* 2019;119:54-60. doi:10.1016/j.maturitas.2018.11.006.
